# Supplementary material for: A high-resolution mRNA expression time course of embryonic development in zebrafish
Source: eLife. 2017 Nov 16;6:e30860. doi: 10.7554/eLife.30860 (PMC5690287; doi:10.7554/eLife.30860)
Supplement: Supplementary file 6. [file elife-30860-supp6.zip › biolayout-clusters-files/Cluster207-genes.html]

Cluster207


# Cluster207: Genes

| Ensembl ID | Gene Name | Chr | Start | End | Biotype |
| --- | --- | --- | --- | --- | --- |
| ENSDARG00000063518 | ENSDARG00000063518 | 2 | 50630 | 53978 | protein\_coding |
| ENSDARG00000035257 | akr1a1a | 5 | 40920886 | 40931476 | protein\_coding |
| ENSDARG00000012076 | apoa1a | 5 | 37378348 | 37381368 | protein\_coding |
| ENSDARG00000103760 | cfhl2 | 22 | 23707437 | 23721720 | protein\_coding |
| ENSDARG00000019713 | oatx | 21 | 44088983 | 44109518 | protein\_coding |
| ENSDARG00000030588 | slc10a1 | 20 | 28958556 | 28964869 | protein\_coding |
| ENSDARG00000000551 | slc1a4 | 13 | 24300770 | 24317047 | protein\_coding |
